# Phenomenology and clinical relevance of minor neurological signs in child neurology and psychiatry

**DOI:** 10.3389/fneur.2026.1761780

**Published:** 2026-05-08

**Authors:** Francesca Magostini, Giorgia Paris, Alessandro Capuano

**Affiliations:** Child Neurology and Psychiatry, Azienda Sanitaria Locale Viterbo, Viterbo, Italy

**Keywords:** minor neurological signs, neurodevelopment, neurological examination, neurological soft signs, psychiatric diseases

## Abstract

Minor Neurological Signs, also referred to as neurological soft signs, are subtle abnormalities detected during neurological examination that do not meet criteria for major focal deficits. They are increasingly considered indicators of variability in neurodevelopment, likely reflecting differences in sensorimotor integration and maturation of cortico–subcortical networks. This mini review summarizes current evidence on the phenomenology, neurobiological correlates, and clinical relevance of MNS in child neurology and psychiatry. MNS include motor features such as overflow movements, dysmetria, dysrhythmia, and mild alterations in coordination, tone, and balance. Their assessment relies on standardized, developmentally appropriate tools that support identification of distinct patterns of dysfunction. MNS are frequently reported in neurodevelopmental and psychiatric conditions. While not diagnostically specific, they have been associated with symptom severity and functional outcomes. Further longitudinal and integrative studies are needed to clarify their developmental trajectories, neurobiological mechanisms, and potential clinical utility.

## 1 Introduction

Minor Neurological Signs (MNS), also called Neurological Soft Signs (NSS), are defined as minimal alterations in the standard neurological examination remaining below the clinical threshold of “major signs.” MNS are thought to be anomalies in sensorimotor integration and cannot be attributed to cerebral focal lesions (1, 2). The debate concerning the clinical significance of MNS evolved concurrently with, and was closely related to, the broader discussion on the conceptualization of minimal brain dysfunction or minimal brain damage (3, 4). With the emergence of modern neurodevelopmental diagnoses, these mild neurological signs began to be increasingly associated with certain neurodevelopmental disorders (NDD) more than others. An indication of this complexity is the lack of a universally accepted label to describe them: in the past, some authors referred to them as equivocal signs (5), others as soft signs or non-focal neurological signs (6), subtle signs (7). Tupper (8) proposed an internal classification within MNS, distinguishing between two subtypes: on the one hand, signs considered to be mild forms of hard neurological signs, such as hypertonia, asymmetrical reflexes, or choreiform dyskinesia; on the other hand, signs described as developmental, reflecting deviations from typical developmental trajectories, including anomalies in diadochokinesia, in finger tapping, or the presence of synkinesis. Among the pioneering studies, those of Hadders-Algra and collaborators (1, 9, 10) described MNS as mild motor abnormalities that cannot be explained by major brain lesions, but rather as expressions of a mild, often diffuse, subcortical brain dysfunction.

Even after many years of investigation, there are still some open questions. One of them concerns the fact that minor neurological signs can be also found in typically developing children and adolescents (11). The persistence of these signs could better correlate with the atypical development, thus considering age a key factor in brain structures maturation.

## 2 Phenomenology

From a phenomenological and clinical standpoint, movement abnormalities in childhood can be framed within the broader classification of movement disorders (12), traditionally divided into hyperkinetic and hypokinetic conditions based on the presence of excessive or reduced involuntary movements. Although this framework has been primarily developed in adult neurology, it provides a useful reference for organizing motor signs observed during developmental neurological examination.

Within this perspective, it is essential to distinguish between major movement disorders, typically associated with well-defined neurological conditions, and MNS, which represent subtle, non-localizing motor abnormalities that may index variability in neurodevelopmental trajectories, rather than reflecting a discrete or static neurological condition.

In line with the movement disorders classification and subsequent classification efforts in the field of minor neurological signs (13), we can identify the following phenomenological categories:

1. *Hyperkinetic movement disorders*: these are characterized by involuntary movements, primarily manifesting as tremors, choreiform movements, and dystonic postures.
  - *Tremor*: defined as a rhythmic, oscillatory movement of a body part, resulting from alternating or synchronous contractions of antagonist muscles, and may occur at rest, during posture, or during action.
  - *Choreiform movements*: frequently described as “dance-like” or “piano playing movements”, consist of brief, irregular, non-rhythmic, and unpredictable movements that flow randomly from one body part to another, predominantly affecting the distal extremities. These movements are not suppressible and are characterized by variability in timing, amplitude, and distribution.
  - *Dystonia*: characterized by sustained or intermittent muscle contractions causing abnormal, often repetitive movements or postures. These movements are typically patterned, twisting, and may be triggered or worsened by voluntary action.
2. *Overflow movements*: these refer to involuntary movements of body parts that are not necessary to perform a motor task effectively. Notable examples include contralateral motor overflow and mirror movements.
3. *Dysmetria*: this is identified as an inability to control the trajectory of purposeful movements, particularly concerning coordination of the extremities.
4. *Miscellaneous disturbances*: this category includes mild alterations in muscle tone, abnormalities in balance and gait (e.g., tandem gait), lateralization and dysrhythmia (an impairment of motor timing and of the ability to generate, maintain, or synchronize temporal sequences of movement, resulting in irregularity in rhythmic execution and coordination).

Although individually non-specific, these signs reflect variations in the organization and integration of distributed sensorimotor networks and are commonly observed within the spectrum of minor neurological signs.

A representative collection of some of those neurological signs is shown in Supplementary Video 1.

Additionally, MNS can be further conceptualized according to the developmental approach proposed by Hadders-Algra et al. (14), which emphasizes the distribution of abnormalities across functional domains rather than the presence of isolated signs. In this model, neurological findings are organized into clusters representing distinct domains of motor and sensory functioning. These clusters typically encompass functional domains such as posture and muscle tone regulation, coordination and balance, fine motor control, associated movements (including overflow phenomena), and sensory-motor integration. Within this framework, individual signs gain clinical relevance not in isolation but in relation to their distribution across these subsystems.

In prepubertal children, classification is primarily quantitative: children are considered neurologically typical when all clusters fall within normal limits, while the involvement of one or two clusters defines simple MNS, and abnormalities affecting multiple clusters define complex MNS.

After puberty, the expression of MNS tends to become more circumscribed, and classification shifts toward a qualitative interpretation based on the nature of the dysfunction. In this phase, simple forms are typically associated with mild abnormalities such as hypotonia or choreiform dyskinesia, whereas complex forms involve higher-order deficits, including impairments in fine motor coordination and motor planning (2, 14).

### 2.1 Clinical assessment

Initiatives aimed at developing standardized neurological examination techniques suitable for assessing mild motor dysfunctions in children commenced in the 1970s and continue today. These evaluations consider typical neurological development and aim to identify functional deficits while confirming normal function or recognizing delays attributed to slower maturation (1).

Various standardized tools are employed for evaluating motor neurodevelopmental skills in children (Table 1), each differing in age range, target of assessment, number of items, administration time, scoring system, output type and application contexts.

**Table 1:** Main scales to assess minor neurological signs.

Tool	Age range	Target of assessment	Items (n) and administration time	Domains/Subscores	Scoring system and output type	Clinical utility for MNS
HINE (Hammersmith Infant Neurological Examination) (20)	2–24 months	Early abnormalities in tone regulation, posture, spontaneous movements, reflexes, cranial nerve function	Item number: 26 Administration time: 5–10 min	5 domains: cranial nerves, posture, movements, tone, reflex reactions	Item-level scoring summed into total score (0–78); cut-offs available Quantitative output	Early detection of atypical neurological trajectories; strong predictive validity for later motor outcomes; not specific for NSS but identifies early precursors
Hempel Examination (based on Touwen) (15)	18 months – 4 years	Early signs of MND, including coordination deficits, tone abnormalities, and postural control issues	Item number: 30–40 (age-adapted) Administration time: ~20–30 min	Age-adapted subsystems derived from Touwen framework	Qualitative assessment with developmental interpretation Qualitative output	Transitional tool bridging infant neurological exam and formal MND assessment; useful for early detection of subtle dysfunctions
PANESS (Physical and Neurological Examination for Soft Signs) (revised versions) (7)	3–12 years	NSS including dyscoordination, motor overflow, balance deficits, and motor sequencing difficulties	Item number: 20–30 (depending on protocol) Administration time: ~20–30 min	Posture, gait, balance, coordination, timed motor tasks, overflow movements	Combination of qualitative ratings and timed quantitative measures Qualitative and quantitative output	Sensitive to NSS in developmental and neuropsychiatric populations (e.g., ADHD, ASD); widely used in research
NESS (Neurological Examination for Subtle Signs, revised) (7)	3–12 years	Subtle neurological abnormalities including motor impersistence, coordination deficits, and minor motor signs	Item number: ~30 Administration time: ~20–30 min	Coordination, posture, reflexes, associated movements, sensory-motor integration	Emphasis on presence/absence and severity of signs Mainly qualitative output	Designed to capture subtle NSS with improved sensitivity; useful in developmental disorders research
ZNA-2 (Zurich Neuromotor Assessment, Second Edition) (19)	3–18 years	Quantitative neuromotor performance (fine/gross motor coordination, speed, variability, and motor control)	Item number: Battery-based (multiple timed tasks) Administration time: ~30–45 min	Fine motor skills, pure motor tasks, static and dynamic balance, associated movements	Norm-referenced z-scores adjusted for age Fully quantitative output	Measurement of motor performance; complements NSS frameworks with objective quantification; suitable for research use
Touwen Neurological Examination (1) (later revisions)	≥4 years	MND (simple vs. complex), including abnormalities in tone regulation, coordination, balance, associated movements, and reflex integration	Item number: 58 Administration time: ~30–45 min	10subsystems into 6 clusters (posture and tone, coordination, fine motor skills, associated movements)	Qualitative classification into MND categories (normal, simple/complex MND) Qualitative (classification-based) output	Gold standard for MND assessment in school-age children; provides clinical classification of NSS patterns and developmental trajectories

The Hempel Examination (15) is tailored for preschool-aged children (18 months–4 years) using a more straightforward assessment approach. In contrast, the Touwen Examination provides those aged 4 years and older with a thorough evaluation encompassing posture, reflexes, coordination alongside fine and gross motor abilities (14, 16). Both PANESS (Physical and Neurological Examination for Subtle Signs) and NESS (Neurological Examination for Subtle Signs) cover similar age groups (approximately 3–12 years) and are adept at detecting subtle deficits in motor skills and balance—especially fine motor skills (7, 17). The Zurich Neuromotor Assessment (ZNA-2), applicable from ages 3 to 18 years, delivers quantitative standardized measurements of neuromotor performance; thus, it proves beneficial for longitudinal studies (18, 19). Touwen and PANESS find frequent use in research environments whereas Hempel and NESS tend toward early clinical identification purposes. While Touwen provides extensive detail but is time-intensive; Hempel offers speed but lacks comprehensiveness; PANESS and NESS necessitate specialized training for reliability; ZNA-2 delivers exact measurements but may be complex in execution. The selection of an assessment tool relies on factors such as the child’s age, assessment goals, along with considerations balancing detail against practicality.

In the context of these assessment tools and with the increasing focus on early detection of neurological signs that may predict later development, recent research has strengthened the role of the Hammersmith Infant Neurological Examination (HINE). HINE is a quick, standardized neurological assessment for infants (2–24 months) that evaluates posture, movements, tone, reflexes, and cranial nerve function to identify early neurological abnormalities and support outcome prediction. Recent studies have strengthened the role of the HINE (20). Romeo et al. showed that HINE can predict outcomes other than cerebral palsy in term infants (21) and that it is useful for longitudinal monitoring of very preterm low-risk infants (22). Dicanio et al. (23) also reported that early neurological assessments can provide long-term predictions of developmental trajectories in low-risk preterm infants, supporting the value of extended follow-up. Finally, Romeo et al. (24) found that gestational age and gender influence early psychomotor development in low-risk preterm infants, suggesting that these factors should be considered when interpreting assessment results.

### 2.2 A continuum model of motor dysfunction

As recently suggested by Hamad and colleagues (25) the concept of Minor neurological dysfunction (MND) does not represent a single, well-defined disorder, but rather a clinical framework describing the distribution and combination of MNS across functional domains. These signs may be found in both typically developing children and those with atypical development, and they can coexist with various neurodevelopmental conditions. MND has been linked to difficulties in motor function, behavior, learning, and cognition. In the motor domain, the deficits associated with MND are frequently considered within the broader category of developmental coordination disorder (DCD), consequently, MND may be particularly relevant to the diagnosis of motor impairments that fall outside cerebral palsy (non-CP motor impairments). Cerebral Palsy (CP) is a group of permanent disorders of the development of movement and posture, causing activity limitation, that are attributed to non-progressive disturbances that occurred in the developing fetal or infant brain (26, 27). In this context, increasing attention has been given to the idea of a phenotypic continuum of motor dysfunction, ranging from clearly defined neurological disorders such as CP to more subtle, non-localizing signs captured under the construct of MNS and MND. Within this continuum, CP represents the severe and clinically overt end, typically characterized by persistent and functionally impairing motor abnormalities associated with identifiable early brain lesions. In contrast, MNS reflect milder and more diffuse alterations, often without clear structural correlates and with a more variable developmental significance. Importantly, while both CP and MNS involve motor abnormalities, they differ in terms of severity, localization, functional impact, and underlying pathophysiology. CP is generally associated with stable, non-progressive lesions of the developing brain and leads to significant activity limitations, whereas MNS are considered markers of atypical neurodevelopmental trajectories, often transient or context-dependent, and not necessarily indicative of a fixed neurological disorder. This distinction is essential to avoid conceptual overlap and to correctly position MNS within the broader framework of developmental neurology.

## 3 MNS: neurobiology behind phenomenology

Two main population-based studies explored MNS in large cohorts of infants from childhood to adolescent (2, 28). Although the Groningen Perinatal Project (GPP) and the following perspective study (2), included not only preterm but also normal birth neonates, the conclusion of Hadders-Algra and collaborators clearly pointed out that perinatal events including low and very low birth weight, are important risk factors for development of later complex MNS, a condition that can be considered a mild form of cerebral palsy. In contrast, simple MNS in otherwise normally developed children should be considered as a delay in the brain maturation. The Étude Épidémiologique sur les Petits Ages Gestationnels (EPIPAGE) project (28, 29), support these observations and highlight prematurity as a key risk factor, with lower gestational age strongly associated with higher MNS rates. Several studies confirmed previous observations (30–34).

On the other hand, simple MNS can occur in children who do not present any perinatal risk factors (35, 36). In such cases, it may be suggested that a typically functioning brain operates in a suboptimal manner. Additional contributing factors have been proposed, including gender, minor perinatal risk factors (such as mild prematurity, low but not very low birth weight, transient perinatal hypoxia, or neonatal complications not leading to overt brain injury) (28, 29), and overarching epigenetic mechanisms. From this developmental perspective, Bonke et al. (37) recently explored changes in brain structure associated with MNS in a cohort of adolescents engaged in regular physical activity. Their research indicated an increase in gyrification within the left superior frontal and parietal regions, along with widespread modifications in white matter microstructure among adolescents exhibiting MNS compared to those without these symptoms. This observation implies a relationship between the MNS phenotype and brain microstructural characteristics. Possible underlying processes might involve changes in synaptic pruning and axonal myelination, which are recognized as key re-wiring events during brain development.

MNS reflect subtle but clinically meaningful aspects of neuromotor development, showing the integrity and maturation of subcortical and cortico–subcortical systems.

Within this neurodevelopmental framework encompassing mild subcortical dysfunction, “soft” (as MNS are considered) and “hard” neurological signs can be understood as lying along a *continuum.* The concept of continuum is a different model where the clinical phenomenology and severity may depend on the degree of involvement of cortico–basal ganglia pathways. Lin and Nardocci (38) elegantly argued that transient, developmental, and pathological forms of dystonia share common pathophysiological mechanisms, rooted in the balance between the motor control and the inhibition of competing motor activity. In this view, one could hypothesize that dystonic postures in childhood can arise from genetic hits, structural lesions (“hard signs”) or subtle disruptions in typical neurodevelopmental trajectories, leading to soft signs as MNS.

It has been demonstrated that identical genetic alterations can lead to a wide range of phenotypic outcomes, spanning classical NDD and more subtle or late-emerging neuropsychiatric conditions (39). These findings support the developmental brain dysfunction model, which proposes that diverse clinical presentations reflect a shared underlying disturbance of brain development, conceptually related to — but broader than — the historical notion of minimal brain dysfunction (40). In this context, motor functioning, often underemphasized in traditional diagnostic frameworks, represents a particularly sensitive indicator of neurodevelopmental disruption, frequently manifesting as delayed motor milestones, impaired coordination, dyspraxia, altered postural control, or subtle neurological signs (41).

Recently, population-based studies (42) using longitudinal birth cohorts indicate that NDD copy number variants (CNVs) are associated not only with later clinical diagnoses but also with early developmental markers, including birth complications and preterm birth, fine and gross motor delay, hypotonia, difficulties in motor coordination, reduced cognitive ability and learning difficulties.

Nevertheless, the rise of genotyping via whole exome sequencing has reshaped the genetic landscape of movement disorders and neurodevelopmental disorders (43). A genetic etiology is demonstrated in more than 30% of cases of cerebral palsy and cerebral palsy mimics, with the most frequent monogenic causes of CP relying on a limited number of neurodevelopmental pathways, notably transcriptional regulation (CTNNB1, FOXG1, MECP2), neuritogenesis (ATL1, KIF1A, SPAST, TUBA1A, TUBB4A), and synaptic transmission (CACNA1A, GNAO1, KCNQ2, SCN1A) (43, 44). Finally, subtle neurological signs or more generally motor difficulties early in the first years of life have been reported especially involving those genes with cellular regulatory properties or contributing to the synaptogenesis (45–47).

## 4 Clinical contexts

MNS are often reported in children and adolescents with neurodevelopmental and psychiatric disorders. Their presence is not in itself diagnostic but may serve as an early warning signal within a comprehensive, multidisciplinary evaluation.

### 4.1 Attention-deficit/hyperactivity disorder (ADHD)

ADHD is a neurodevelopmental disorder defined by impairing levels of inattention, disorganization, and/or hyperactivity-impulsivity (48). MNS are highly prevalent in children with ADHD, with studies consistently reporting occurrence rates between 80–93% compared to substantially lower rates in typically developing controls (49). This elevated prevalence reflects the core neurodevelopmental character of the disorder, supporting the hypothesis that ADHD is etiologically related to delayed brain maturation (50). Motor deficits including dysrhythmia, overflow, and slow speed of timed activities represent the most common MNS categories in ADHD (51–54). These motor abnormalities appear across all ADHD subtypes, though certain signs show differential presentation depending on subtype classification. Children with ADHD show significantly more dysrhythmia in timed movements and slower speed during repetitive finger and hand tasks compared to typically developing peers (55). Motor overflow - excessive unnecessary movements during motor tasks - is markedly elevated in children with ADHD, particularly in males during childhood, though this sign shows notable developmental improvement with age (54, 55). Specifically, boys with ADHD exhibit greater reductions in overflow from childhood to adolescence than typically developing boys, suggesting that motor overflow may resolve through developmental maturation in males while other motor signs persist. Fine motor deficits in grip strength and manual dexterity are commonly observed, alongside gross motor coordination difficulties affecting gait, balance, and postural stability (56). MNS severity correlates with overall ADHD symptom severity and functional impairment, suggesting these motor signs reflect core aspects of ADHD pathophysiology rather than incidental comorbidity. Furthermore, the correlation between MNS and executive functions implicates neural circuits supporting response inhibition and motor control, with specific motor signs demonstrating differential predictive validity for distinct inhibitory processes. Performance on executive function measures including the Stroop Color-Word Test correlate significantly with total overflow in timed movements, suggesting that motor inhibition deficits reflect broader executive dysfunction (56, 57). The common neurobiology may rely on dysfunctions in dopaminergic and frontostriatal circuits, as demonstrated by neuroimaging and genetic studies (58–62). Additionally, alterations in brain-derived neurotrophic factor (BDNF), which has a critical role in neuronal development (63), have been implicated in various neurodevelopmental disorders. BDNF emerges as a key factor in understanding the interplay between MNS and executive functions in ADHD, particularly given the reported BDNF abnormalities (64–66). Tunagur et al. (57) suggest that the association between MNS, visuospatial abilities, and selective attention may reflect a maturational delay in ADHD pathophysiology, highlighting a potential role of BDNF in this developmental lag. Consistently, Pitzianti et al. (67) reports that individuals with ADHD frequently exhibit an increased number of overflow movements (OMs), impaired timing of motor responses, deficits in motor coordination, and fine motor impairments. Specifically, OMs are thought to reflect dysfunction within motor and premotor circuits, dysrhythmia may indicate cerebellar involvement, and slowness in timed tasks may result from functional deficits in frontostriatal networks, the cerebellum, and basal ganglia structures (68, 69). The association between inattentive symptoms and neurological signs is further supported by pharmacological studies with methylphenidate (MPH). In children with ADHD, MPH treatment is associated with improvements in attention and executive function deficits, as well as notable improvements - or even complete resolution - of MNS (70). These findings suggest that MNS evaluation could serve as a useful measure for monitoring MPH treatment efficacy in ADHD.

### 4.2 Autism spectrum disorder (ASD)

Autism spectrum disorder (ASD) is a neurodevelopmental condition primarily characterized by social and behavioral impairments, as well as restricted, repetitive, and stereotyped interests (48). Over the past two decades, several studies have documented motor delays and anomalies in motor coordination among children and adults with ASD (71–74). In a comprehensive study examining children with autism without intellectual disability, researchers found that 96.9% of children with ASD had minor neurological dysfunctions compared to only 15.6% of healthy controls (75). Within the ASD group, 81.3% demonstrated simple minor neurological dysfunctions, while 15.6% exhibited complex forms. The specific types of neurological abnormalities most frequently observed in children with autism include fine manipulative disability, sensory deficits, and choreiform dyskinesia, along with an excess of associated movements and anomalies in coordination and balance (76). Motor impairments often represent some of the earliest observable symptoms, preceding core social and communicative deficits, and tend to persist and exacerbate the clinical presentation (77–79). More recently, Wieting et al. (80) investigated MNS and motor skills in adults with high functioning autism, reporting that these individuals differ from healthy controls primarily in MNS related to motor coordination. Overall, these findings indicated that MNS have high diagnostic value in distinguishing patients from typically developing individuals. These observations suggest that careful analysis of motor signs can provide specific insights in ASD, as motor, social, and communicative abnormalities may reflect correlated traits arising from dysfunctions in parallel neural circuits.

### 4.3 Developmental coordination disorder

Developmental Coordination Disorder (DCD) is characterized by motor performance in activities requiring coordination that is substantially below what is expected for a child’s age and intelligence, and which interferes with academic achievement or activities of daily living (81). Children showing MNS frequently fall within the broader category of DCD, and a relationship has been highlighted in numerous studies (82, 83). Thus, neurological examination may be a valuable diagnostic tool for children with motor difficulties including DCD, and it should specifically assess the presence of MNS (84). Unfortunately, a limited number of papers (85–88) have assessed the co-occurrence of MNS in DCD patients using standardized assessment tools such as M-ABC (Movement Assessment Battery for Children – 2nd edition) for DCD and one of the above-mentioned scale for MNS. Overall, findings suggest that children with complex MNS often score below the threshold for DCD on the M-ABC, experiencing motor difficulties that interfere with everyday functioning. Peters et al. (84) discussed minor neurological dysfunction, emphasizing that the diagnosis relies on coherent clusters of signs rather than individual symptoms, which gain clinical significance only when co-occurring within a functional domain. The study demonstrates that poor motor performance at school age was associated with more severe neurological dysfunction (complex MNS), particularly affecting fine motor manipulation and coordination. This association was especially evident in children whose coordination test scores were significantly below average. More recently, Sueda et al. (89) examined the relationship between coordination tests and questionnaires and the presence of MNS, finding a strong positive correlation between test scores and MNSscores, further supporting a link between motor impairments and neurological signs. MNS carries important implications for clinical assessment, diagnosis, and treatment. According to the European Academy of Childhood Disability (81), children with DCD frequently show signs of neurodevelopmental immaturity that overlap with MNS, including involuntary movements of unsupported limbs, mirror movements, and deficits in both fine and gross motor skills. Recognizing and classifying MNS in children with DCD is therefore crucial, as it can guide both intervention strategies and help inform prognosis.

### 4.4 Psychiatric disorders: obsessive-compulsive disorder (OCD), bipolar disorder, first-episode psychosis

In addition to neurodevelopmental disorders, MNS are found across psychiatric conditions. Jaafari et al. (90) conducted a meta-analysis in obsessive-compulsive disorder (OCD) and found greater presence of MNS than controls - particularly in motor coordination, sensory integration, and primitive reflexes - which supports neurobiological mechanisms of development beyond purely psychological causes. Familial aggregation studies (91) indicate that first degree relatives may display similar signs, and that a higher burden of MNS has been observed in individuals with OCD presenting comorbid psychotic features. This finding suggests that MNS may index increased neurobiological vulnerability in specific OCD subgroups characterized by reduced insight and greater clinical severity. Furthermore, MNS have been correlated with poor insight and cognitive deficits, further supporting their application in screening clinical phenotypes (92). In schizophrenia spectrum disorders, a recent study (93) showed that MNS are closely associated with cognitive deficits and parkinsonian symptoms. In Bipolar Disorders, meta-analytic evidence suggests the presence of elevated MNS in comparison with controls, which is generally less severe compared to schizophrenia; thus, supporting a transdiagnostic interpretation of MNS across psychiatric disorders (94). Studies suggest that MNS have a correlation with both the course of the illness and psychosocial functioning, paving way in determining their usefulness as staging markers (95, 96). Systematic reviews of first-episode psychosis (FEP) (97) find that motor abnormalities like MNS, parkinsonism and dyskinesia correlate with symptom severity, brain changes and functional decline over time. This highlights the need for the proper assessment of disease, for early intervention, clinical rehabilitation work-up and prognosis. They are particularly useful for identifying subgroups in OCD (especially low-insight patients) and in BD to assess functional outcomes, and finally serving as early markers in FEP.

## 5 Conclusion

The systematic observation of MNS provides clinically relevant information on the functional organization of developing neural systems involved in motor control and sensory integration. Rather than directly informing etiology, MNS can be understood as observable markers of variability in the organization and functioning of subcortical and cortico–subcortical systems. However, their clinical relevance is often underestimated and not always fully recognized during assessment, despite their potential to support early identification of risk in children who do not yet meet full diagnostic criteria but present emerging signs of neurodevelopmental vulnerability.

In the clinical context, MNS should be conceptualized as structured, domain-specific configurations of signs that support the identification of neurodevelopmental subgroups. MNS can be interpreted as intermediate phenotypes along a spectrum that includes, on one end, transient maturational variations and, on the other, conditions characterized by structural and persistent deficits, such as cerebral palsy.

**Figure 1:**
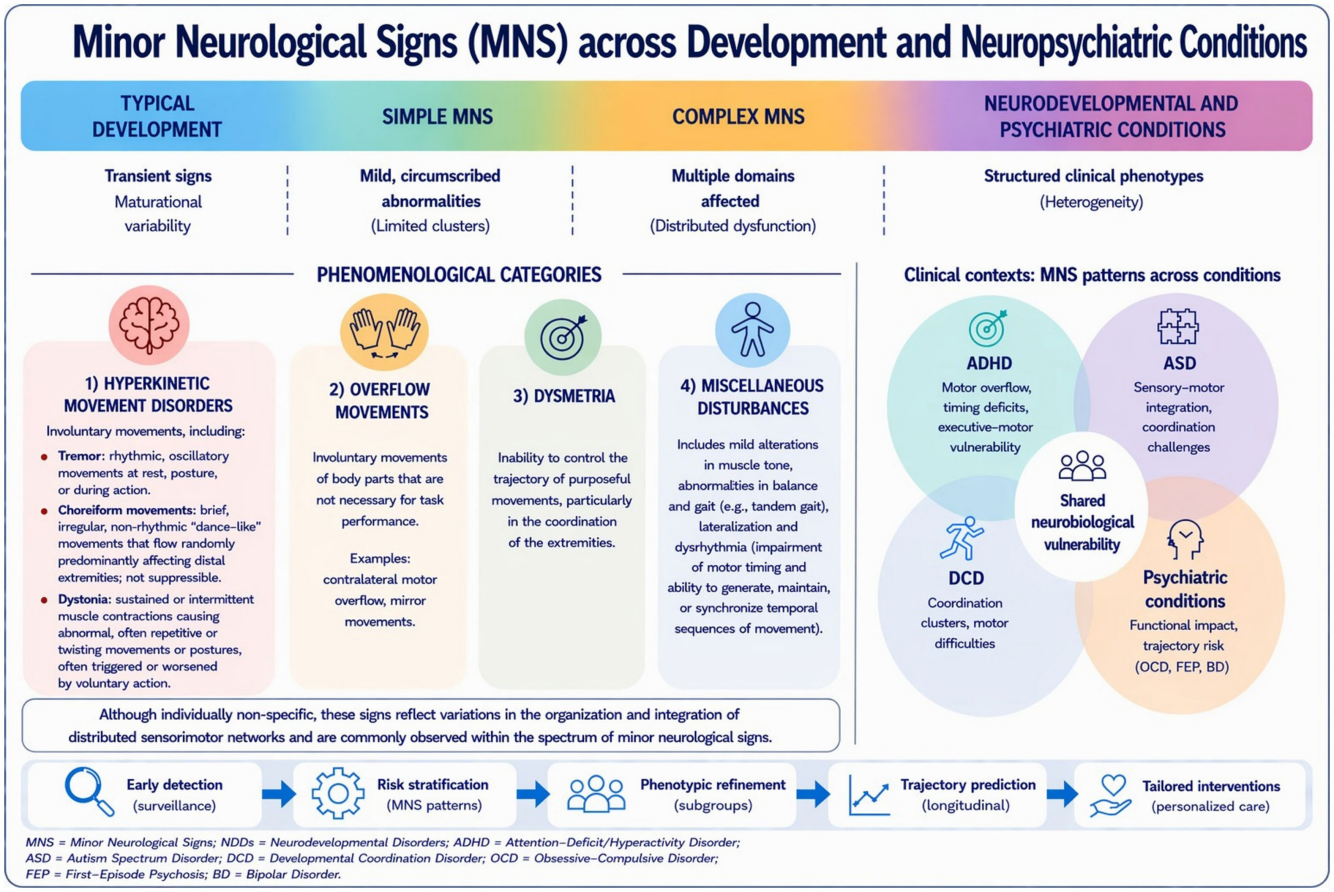
Minor neurological signs (MNS) across development and neuropsychiatric conditions. Schematic representation of MNS along a continuum from typical development to neurodevelopmental and psychiatric disorders. The phenomenology of MNS in the clinical setting shows partial overlap across conditions (e.g., ADHD, ASD, DCD), suggesting shared neurobiological vulnerability and supporting their role in early detection and clinical stratification.

The systematic assessment of MNS using standardized and specific tools is essential to clinical practice. Recognizing MNS as central components of neurodevelopmental assessment may improve early diagnosis, refine phenotypic stratification, and support the implementation of earlier and more tailored interventions in neurodevelopmental disorders (Figure 1).

Future studies should prioritize longitudinal designs to clarify developmental trajectories and the prognostic value of MNS.

## Supplementary material

SUPPLEMENTARY VIDEO 1 Phenomenology of MNS. The video complements the description provided in the manuscript by showing MNS more frequently encountered in the clinical practice during standard neurological examination. Segment 1: both arms outstretched in front; Segment 2: tapping (finger and hand tapping); Segment 3: tandem gait.
