# Supplementary material for: Phenomenology and clinical relevance of minor neurological signs in child neurology and psychiatry
Source: Front Neurol. 2026 May 8;17:1761780. doi: 10.3389/fneur.2026.1761780 (PMC13193831; doi:10.3389/fneur.2026.1761780)
Supplement: Supplementary file 2 [file Table_1.DOCX]

Video 1 Caption

Phenomenology of MNS. The video complements the description provided in the manuscript by showing MNS more frequently encountered in the clinical practice during standard neurological examination. Segment 1: both arms outstretched in front; Segment 2: tapping (finger and hand tapping); Segment 3: tandem gait.
